# Robotic Radical Cystectomy: Where are We Today, Where will We be Tomorrow?

**DOI:** 10.1100/tsw.2010.217

**Published:** 2010-11-16

**Authors:** Kyle A. Richards, A. Karim Kader, Ashok K. Hemal

**Affiliations:** Department of Urology, Wake Forest University Baptist Medical Center, Winston-Salem, NC, USA

**Keywords:** robotic radical cystectomy, bladder cancer, robotic surgery, radical cystectomy, laparoscopic surgery

## Abstract

While open radical cystectomy remains the gold-standard treatment for muscle-invasive bladder cancer and high-risk non–muscle invasive disease, robotic assisted radical cystectomy (RARC) has been gaining popularity over the past decade. The robotic approach has the potential advantages of less intraoperative blood loss, shorter hospital stay, less post-operative narcotic requirement, quicker return of bowel function, and earlier convalescence with an acceptable surgical learning curve for surgeons adept at robotic radical prostatectomy. While short to intermediate term oncologic results from several small RARC series are promising, bladder cancer remains a potentially lethal malignancy necessitating long-term follow-up. This article aims to review the currently published literature, important technical aspects of the operation, oncologic and functional outcomes, and the future direction of RARC.

## INTRODUCTION

Robot-assisted radical cystectomy (RARC) with urinary diversion and bilateral pelvic lymph node dissection (PLND) for the management of muscle-invasive bladder cancer and high-risk superficial bladder cancer has been gaining popularity over the past 8 years. As urologists have become more comfortable using the robotic platform for radical prostatectomy, institutions across the U.S., Europe, and Asia have been performing this increasingly complex robotic operation (RARC + PLND) with acceptable operative, perioperative, and short-term oncologic outcomes. This article aims to review the currently published literature, discuss the important technical aspects of the operation, examine oncologic and functional outcomes, and will touch on the future direction of RARC.

## OPERATIVE TECHNIQUE (WAKE FOREST UNIVERSITY TECHNIQUE OF RARC)

The initial description of the three-step technique of RARC in men and women using six ports (three for robotic instruments and three for bedside assistance), with extracorporeal reconstruction of ileal conduit (ECRIC) or orthotopic ileal neobladder (ECRONB), was first made by Menon and Hemal, and serves as the foundation for subsequent modifications and evolution of the surgical procedure[1,2]. With the advent of the four-arm da Vinci S platform, one of the assistant ports is replaced with a robotic arm, leaving the remaining two assistant ports for the critical bedside assistant. Additionally, since the initial descriptions in 2003, several other authors have reported feasibility and success with subtle modifications of RARC with urinary diversion and PLND[3,4,5,6].

## Wake Forest University Technique of RARC in Males

The three major steps of the operation include radical cystoprostatectomy, bilateral PLND, and urinary diversion. The authors prefer to perform ECRIC or ECRONB with robot-assisted urethral-neobladder anastomosis; however, intracorporeal urinary diversion is technically feasible as demonstrated by several groups[7,8,9]. The sequence of steps may vary from surgeon to surgeon.

### *Step 1. Preparations and Port Placement*

General anesthesia is achieved, and both a gastric tube and Foley catheter are inserted. The patient’s arms are adducted and padded, and the table is placed in the steep Trendelenburg position (20–45°). Pneumoperitoneum is created using a Veress needle (Ethicon Endo-Surgery, Albuquerque, NM) through a vertical skin incision approximately 3–4 cm above the umbilicus. Early on in one’s learning curve, it may be useful to make these measurements with a ruler and mark out the planned port sites on the deflated abdomen to help assist in the critical placement of the ports and to avoid robotic arm collisions that may occur from poor port location. The abdomen is insufflated to 15–20 mmHg and the 12-mm camera port is placed. The abdominal cavity and pelvis are inspected, locating any adhesions or obvious metastatic disease. The remaining five ports are placed under direct laparoscopic vision using carefully measured specifications to ensure a successful operation, especially an adequate PLND, and for transfer of the left ureter to the right under the sigmoid mesentery (see Fig. 1). The first two robotic ports are placed 10 cm lateral inferior to the camera port and 17 cm from the mid-line of the superior aspect of the pubis. The third robotic port is placed 10 cm lateral to the left-sided robotic port, three fingerbreadths superior to the left anterior, superior, iliac spine (ASIS). A 12-mm assistant port is placed 10 cm lateral to the right-sided robotic port, three fingerbreadths superior to the right ASIS, and a final 12-mm assistant port is placed through a premarked site for the stoma of the ileal conduit. The robot is then docked between the patient’s legs. During the procedure, we typically utilize the monopolar curved scissors (right hand), plasmakinetic bipolar forceps (*Gyrus ACMI PK*™) in the left hand, and a grasper such as the *ProGrasp*™ forceps in the fourth robotic arm. We prefer to use the 0° lens; however, we utilize the 30° lens for parts of the dissection deep in the pelvis when needed.

### *Step 2. Adhesiolysis*

All adhesions are taken down using a combination of blunt and sharp dissection, with electrocautery used sparingly. The bedside laparoscopic assistant is critical in providing countertraction using a blunt grasper and a suction tip irrigator.

### *Step 3. LN Dissection*

The peritoneum is incised over the external iliac artery distally to the femoral canal with the aid of the bedside assistant’s countertraction on the peritoneal fold, thus exposing the retroperitoneal space and its relevant anatomical landmarks. The fourth arm *ProGrasp*™ forceps are critical during the PLND for retraction of the sigmoid colon to gain adequate exposure especially when doing the proximal dissection

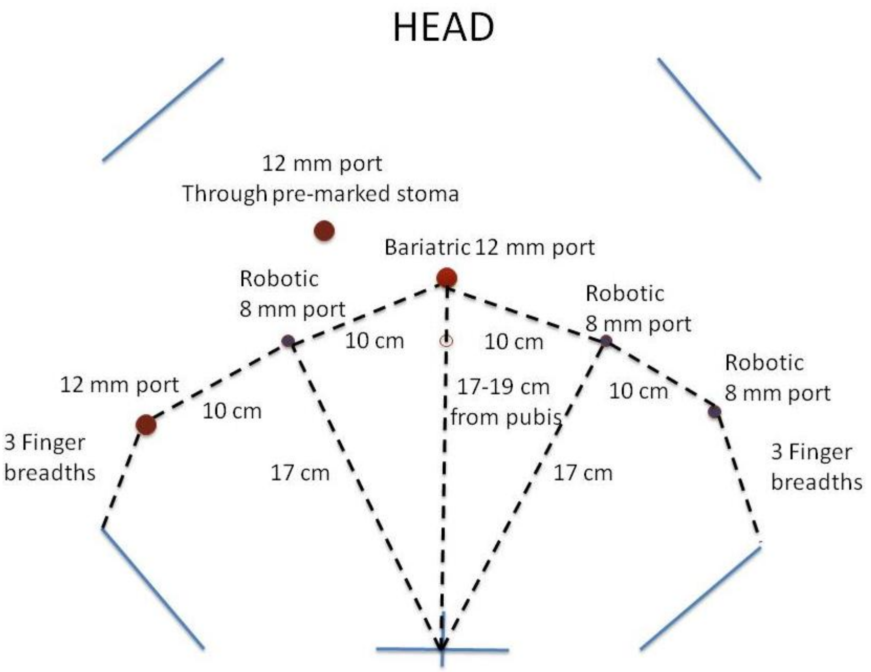

**FIGURE 1.** Port placement for the da Vinci S model.

close to the bifurcation of the aorta. Plasmakinetic bipolar forceps and Hem-o-lok clips are used to control lymphatic channels throughout the dissection.

## Standard LN Dissection

The first step is to identify the pulsations of the external iliac artery. The external iliac artery and vein may not be readily identifiable, especially in patients with a large amount of adipose tissue; however, they can be located by visualizing the pulsations in their usual anatomic position. These vessels are isolated using blunt dissection with the suction tip irrigator or the closed monopolar scissor tips. The external iliac vein is located posterior and slightly medial to the external iliac artery (see Fig. 2). Identifying the correct avascular fibroalveolar plane of dissection above the artery and vein is critical. The obturator and hypogastric packets are prepared by identifying the medial border of the external iliac vein (see Fig. 3). The nodal tissue is carefully dissected away from the vein distally to the pubic bone and the node of Cloquet. Importantly, the circumflex vein and other aberrant tributaries of the external iliac vein or obturator vein can be encountered at the distal-most aspect of this dissection. Due to the pneumoperitoneum, the veins may be compressed and thus more susceptible to injury. To facilitate this dissection, the bedside assistant can retract the external iliac vein laterally, while the surgeon provides countertraction on the obturator packet medially. In addition, the pneumoperitoneum can be lessened to 10 mmHg to minimize venous compression. A combination of blunt dissection, release of fibrofatty attachments using monopolar scissors, bipolar cauterization of larger vessels and lymphatics, and Hem-o-Lok clips as needed results in a thorough LN dissection. The distal margin of the LN packet is the pubic bone just anterior to the obturator nerve and vessels, with the proximal end of the dissection being the mid-portion of the common iliac artery. It is important to note that the ureter often passes over the bifurcation or distal end of the common iliac artery, and careful attention must be made to not injure this critical structure. As the obturator packet is divided distally, including the node of Cloquet, it is peeled back cephalad. To achieve exposure for the hypogastric packet, the median umbilical ligament should be

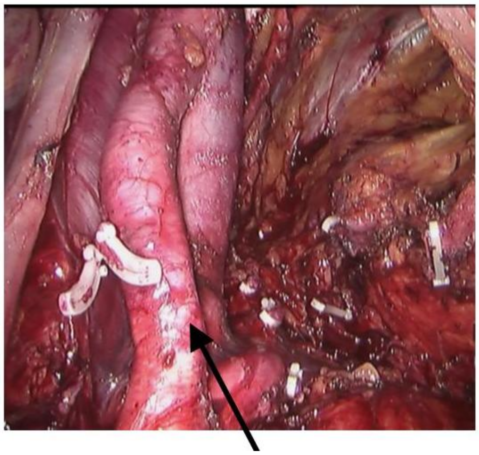

**FIGURE 2.** External iliac vessels.

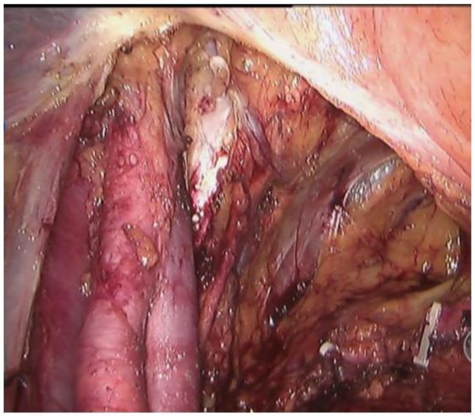

**FIGURE 3.** Obturator LN.

retracted medially and the external iliac vein should be kept in view. It is important to note that the internal iliac artery does not have the same fibroalveolar sheath as the external iliac artery and the nodal tissue may be more adherent. This may necessitate more sharp dissection and ligation of small blood vessels prior to division of the packet at the level of the bifurcation of the common iliac artery. In summary, the limits of dissection are the following: the genitofemoral nerve laterally, bladder wall medially, inguinal ligament distally, and pelvic floor and hypogastric vessels inferiorly.

## Extended LN Dissection

An extended LN dissection includes a standard LN dissection plus the following. Starting at the bifurcation of the aorta and progressing distally down the common iliac artery, all the nodal tissue is dissected free, making sure to skeletonize these vessels (see Figs. 4 and 5). Using the four-arm da Vinci S or Si model, it is not necessary to alter port placement if planning to perform an extended LN dissection as the robotic arms have longer excursion; however, if one is using the standard platform, it may be necessary to place the robotic ports 2 cm cephalad in order to technically be able to perform this dissection. The ureter should be identified, dissected, isolated, and tucked high into the peritoneal cavity prior to embarking on the LN dissection.

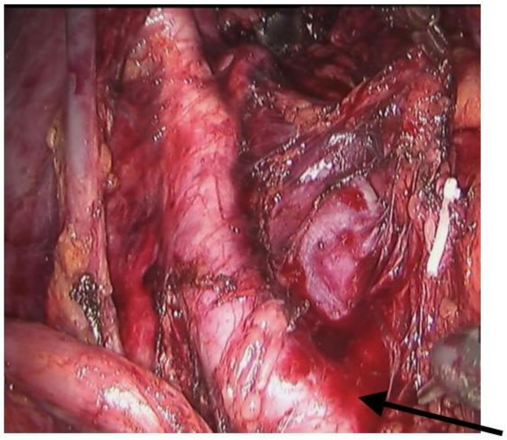

**FIGURE 4.** Bifurcation of aorta.

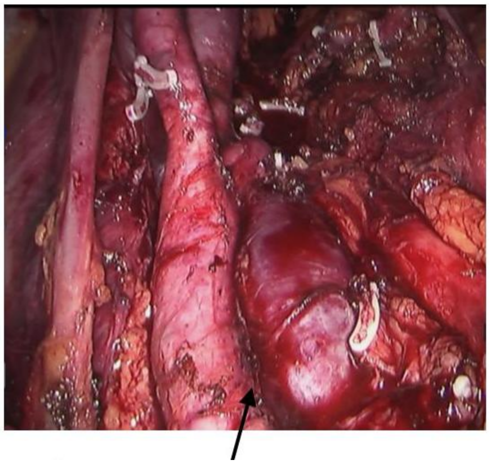

**FIGURE 5.** Common iliac vessels.

The procedure is performed bilaterally and the LN are retrieved using a grasping device and sent in separate packets when possible or the nodal packages are placed in EndoCatch I® bags (United States Surgical Corporation, Norwalk, CT) for LN retrieval and prevention of port-site recurrence. After completion of the node dissection, aggressive sterile water irrigation is carried out and thorough hemostasis is achieved.

## *Step 4. Radical Cystectomy*

### Robot-Assisted Radical Cystoprostatectomy in Males

Bilateral ureters are mobilized and the distal ends of the ureters are divided and sent for frozen section analysis. A Hem-o-lok clip is applied just proximally to prevent urine spillage and to allow for ureteral dilation to assist in dissection and later ureterointestinal anastomosis. The posterior dissection is aided with the 30° down lens. The peritoneum is incised transversely in the cul-de-sac anterior to the rectum. The vasa deferentia are identified and dissected to mobilize the ampulla of the vas, seminal vesicles, prostate, and base of the bladder off the anterior wall of the rectum. The dissection is carried out laterally to identify the prostatic and vesical pedicles that can be ligated with separate Hem-o-lok clips or with sequential firing of the articulating endoscopic gastrointestinal anastomosis (GIA) stapler (Ethicon Endo-Surgery or Endo GIA™, Covidien, Mansfield, MA). This dissection is carried forward and neurovascular bundles can be preserved in select individuals.

The urachus and medial umbilical ligaments are divided to drop the bladder and enter into the space of Retzius. The endopelvic fascia is incised bilaterally and the dorsal venous complex is controlled with a stitch or stapling device. Note that use of the stapling device obviates the need for use of needle drivers for the male patient. The urethra can then be divided and the Foley catheter secured with an extra-large Hem-o-lok clip before dividing the catheter to prevent urine and tumor spillage. The specimen, now freed, can be placed into an extra-large 15-mm Endocatch-II bag (United States Surgical Corporation) that is typically placed through the lateral assistant port.

## RARC in Females

While the overall technique, port placement, and patient preparation are similar for women, there are subtle differences that deserve mention. Several groups have reported these technical nuances ranging from vaginal sparing, uterus sparing, and nerve sparing, to conventional robot-assisted anterior pelvic exenteration with operative steps varying based on surgeon preference[2,6,10,11]. A thorough review of robot-assisted surgery in the female pelvis was recently published[12].

The authors approach RARC in females systematically with variations based on location and stage of disease. The ureters are mobilized and ligated along with the superior vesicle pedicles as previously described. A thorough extended PLND is typically performed. To complete the posterior dissection, the posterior wall of the vagina is elevated with the fourth robotic arm and dissection is carried caudally in the rectovaginal space. The ligamentum teres is divided and the anterior dissection is performed as the bladder is dropped. The inferior and middle vesicle pedicles are controlled as previously described. Finally, the specimen is freed by dissecting the urethrovaginal space and with division of the urethra, the specimen can be delivered via the vagina. The vagina is subsequently closed with a running suture in a clamshell fashion. Furthermore, robot-assisted anterior pelvic exenteration has been described in detail by three separate centers with adequate short-term results[6,10,11].

## *Step 5. Urinary Diversion*

The authors typically prefer to perform either ECRIC or ECRONB; however, it is feasible to perform a totally intracorporeal urinary diversion as was nicely described in a recently published atlas by Pruthi and colleagues[7]. One of the earliest descriptions of RARC in 2003 involved a totally intracorporeal orthotopic neobladder creation; however, due to the long operative times and technical difficulty associated with intracorporeal diversion, RARC did not gain popularity until RARC with extracorporeal urinary diversion was described by Menon et al.[1] and later by Hemal and colleagues[13,14]. The extracorporeal diversion is performed through a 5-cm midline incision to the left of the umbilicus. The specimen is also retrieved through this incision. Ileal conduit or continent catheterizable stoma creation can then be performed in a standard open fashion through a mini-laparotomy incision. For ECRONB, once the pouch is constructed extracorporeally, it is placed back in the pelvis, the robot may be redocked, and the neobladder-urethral anastomosis is carried out. Alternatively, interrupted urethral anastamotic sutures may be placed with the assistance of the robot prior to undocking.

In 2004, Balaji and colleagues described their initial results and technique for intracorporeal ileal conduit urinary diversion with RARC in three patients[8]. Total operative time was comparatively long at an average of 11.5 h, but average blood loss and mean hospital stay were 250 mL and 7.3 days, respectively. Sala and colleagues described their technique in a single patient who underwent RARC and total operative time, including intracorporeal ileal orthotopic neobladder creation, was 12 h[9]. Most recently, Pruthi and coworkers, a group with much experience in RARC, published their technique and results for intracorporeal ileal conduit urinary diversion (n = 9) and intracorporeal orthotopic ileal neobladder (n = 3)[7]. In their experienced hands, mean operative time was considerably shorter than other's experiences at 5.3 h; likewise, mean blood loss and mean hospital stay were equally impressive at 221 mL and 4.5 days, respectively. Therefore, in select patients with an experienced team, total intracorporeal urinary diversion may be a reasonable option to consider.

## DISCUSSION

At the time this review was written, RARC has been performed for over 7 years, but has yet to gain widespread acceptance at academic and community centers. However, it has been estimated that 70–85% of the radical prostatectomies performed in the U.S. in 2009 involved the use of robotic assistance[15]. Why is there such a disparity in the use of robotic assistance for prostatectomy compared to cystectomy? One possible explanation for the lack of RARC in community hospitals could be the cost and lack of technical expertise associated with this complex and lengthy operation. It has been well documented that the average community urologist does not perform a large volume of cystectomies per year, which would be problematic not only for the learning curve, but might also be cost prohibitive for the institution. Furthermore, early critics of RARC have been skeptical with regards to the lack of long-term oncologic and functional outcomes[16]. The extent of the robot-assisted PLND has also been criticized, but data are emerging from multicenter studies as well as single-center reports revealing comparable LN yields and medium-term oncologic outcomes for RARC. The proposed benefits of RARC include less intraoperative blood loss, potential for quicker return of bowel function, shorter hospital stay, quicker postoperative convalescence, and shorter learning curve compared to pure laparoscopic surgery.

### Experience from Different Centers: Comparison of RARC with Laparoscopic Radical Cystectomy and Open Radical Cystectomy

After the initial reports surfaced describing the technique and feasibility of RARC, many centers began reporting their single-center experience with perioperative and short-term outcomes (see Table 1). Rhee and colleagues prospectively compared the results in 30 patients with clinically localized bladder cancer who underwent either open radical cystectomy (ORC) with ileal conduit urinary diversion (n = 23) or RARC with ECRIC (n = 7)[17]. The results from this single-institution series revealed a significant decrease in estimated blood loss (EBL) in the RARC group compared to the ORC group (479 vs. 1109 mL, $p = 0.002$), but a significant increase in total operative time in the RARC group (638 vs. 507 min, $p = 0.005$). There was no difference in average hospital stay, but more patients in the ORC group had extravesical disease on final pathology (13 vs. one patient in the RARC group), suggesting the potential for selection bias inherent in these types of analyses. Pruthi and Wallen retrospectively compared their initial experience with RARC and extracorporeal urinary diversion in 20 men to a control group of gender-matched men who underwent ORC (n = 24) during the same time period at their institution[3]. A majority of the patients had cT2 bladder cancer and one-half of the patients underwent ECRONB with the other half undergoing ECRIC. These authors reported acceptable perioperative outcomes, including mean EBL 313 mL and mean total OR time 6.1 h, with 80% of patients being discharged to home on postoperative day 4. There were no positive margins reported and mean LN yield was 19.

Abraham and coworkers went on to compare a single-center experience with laparoscopic radical cystectomy (n = 20) to RARC with ECRIC (n = 14)[18]. The operative times were similar between the two groups; however, there was less blood loss and fewer transfusions in the RARC group. Bilateral extended PLND was performed in the majority of patients with a nonstatistically significant trend towards higher LN excision in the RARC group (22.3 vs. 16.5 nodes, $p = 0.09$). The authors concluded that RARC

**TABLE 1**
**Comparison of RARC Series**

| Authors | # of patients | # LN | Pos Margins | EBL | OR time | Hospital Stay | IC^ | NB" | CC~ |
|---|---|---|---|---|---|---|---|---|---|
| Wake Forest* | 58 | 18 mean 5-45 range | 3% | 415 mean 300 median | 390 min mean** | 7 d mean | 55 | 2 | 1 |
| Pruthi[28] | 100 | 19 mean 8-40 range | 0 | 270 mean 250 median | 4.6 h mean 4.3 h median | 5 d in 85% | 61 | 38 | 0 |
| Wang[19] | 33 | 17 median 6-32 range | 6% | 400 median (100-1200) | 390 min median | 5 d median 4-18 range | 17 | 12 | 3 |
| Murphy[20] | 23 | 16 median | 0 | 278 mean | 397 min mean | 11.6 d mean | 19 | 4 | 0 |
| Rhee[17] | 7 | NR | 0 | 479 mean | 638 min mean | 11 d mean | 7 | 0 | 0 |
| Ng[36] | 83 | 16 median 0-56 range | 7% | 460 mean | 6.25 h mean | 5.5 d median | 47 | 26 | 10 |
| Menon[1] | 17 | NR | 0 | 150 mean | 260-308 min mean*** | NR | 3 | 14 | 0 |
| Guru[23] | 47 | 18 mean 6-43 range | 13% | 520 mean | NR | NR | NR | NR | NR |
| Woods[5] | 27 | 12 mean 7-20 range | 7% | 277 mean | 400 min mean | NR | NR | NR | NR |

^ Ileal conduit
" Orthotopic neobladder
~ Continent cutaneous
*Excludes patients with pT4 disease
**Most recent 25 cases, includes patient repositioning, urinary diversion, and closure
*** 260 for RARC + ECRIC and 308 for RARC + ECRONB
NR = Not reported vs. laparoscopic radical cystectomy appears to have a shorter learning curve, less blood loss, fewer complications, and earlier return of bowel function.

As the technology continued to improve, a four-arm robotic platform called the da Vinci® S™ Surgical System was introduced by Intuitive Surgical (Sunnyvale, CA) in January 2006, which features fast setup, rapid instrument exchange, and multi-image display capabilities. The initial results using the second-generation device were published shortly thereafter by Hemal and colleagues in a case series of six patients with invasive bladder cancer who underwent RARC with either ECRIC (n = 5) or sigmoid ECRONB (n = 1). The perioperative outcomes using the da Vinci® S™ were acceptable, with average operative time, EBL, and hospital stay of 5.5 h, 200 mL, and 9.2 days, respectively; furthermore, the authors reported pathologic outcomes including no positive surgical margins and an average LN yield of 12[13]. The added benefits of the fourth robotic arm include superior bladder retraction, improved ability to define the vascular pedicles and separate the cystectomy specimen off the rectum, and it obviates the need for a second assistant. The robotic instruments are also longer, which aids in performance of an extended PLND.

Wang et al. reported prospectively collected data from a single-surgeon series of 54 consecutive patients who underwent either RARC (n = 33) or ORC (n = 21)[19]. As in previous reports, the robotic cohort had decreased blood loss (400 vs. 750 mL, $p = 0.002$) and transfusion requirements, but increased operative duration (390 vs. 300 min, $p = 0.007$). The robotic group had earlier return of bowel function by 1 day (4 vs. 5 days, $p = 0.002$) and earlier time to discharge by 3 days on average (5 vs. 8 days, $p = 0.007$). In regards to pathologic outcomes, there were significantly more patients with extravesical (57 vs. 28%, $p = 0.03$) and node-positive disease (34 vs. 19%, $p = 0.04$) in the ORC group, suggesting that there was a bias towards open surgery in patients with more advanced disease. Finally, there were two patients in the RARC group and three in the ORC group with positive surgical margins, and the median LN yields were similar in the RARC compared to the ORC group (17 vs. 20, $p = 0.6$). Murphy and colleagues went on to report the initial experience with RARC, PLND, and extracorporeal urinary diversion in 23 patients with bladder cancer in the U.K., revealing a mean operative time, EBL, and hospital stay of 397 min, 278 mL, and 11.6 days, respectively[20]. Interestingly, the average hospital stay for ORC in the U.K. is 18.7 days. Additionally, no positive surgical margins were encountered and the average LN yield was 16, leading the authors to conclude that RARC is an evolving procedure that carries an acceptable morbidity with good short-term oncologic results.

While single-center case series can offer some value to the literature, these studies are often fraught with selection bias inherent in their design. Nix and colleagues set out to minimize bias and provide level one evidence in a randomized prospective clinical trial comparing RARC to ORC at their institution[21]. This study accrued 41 patients, of which 21 were randomized to RARC and 20 to ORC with the primary end point being LN yield with a noninferiority margin of four LN. The RARC patients had significantly lower mean EBL (258 vs. 575 mL, $p < 0.001$), shorter mean time to flatus (2.3 vs. 3.2 days, $p = 0.0013$), and less use of narcotic pain medicine, whereas the RARC patients had a significantly longer operation on average (4.2 vs. 3.5 h, $p < 0.001$). There was no statistical difference in the overall hospital stay (RARC 5.1 vs. ORC 6.0 days, $p = 0.24$) or complication rate (RARC 33 vs. ORC 50%, $p = 0.28$) between RARC and ORC, however there was a trend in favor of RARC with respect to both outcomes. The mean LN yield was similar for the RARC group compared to the ORC group (19 vs. 18, $p = 0.52$); thus, the authors concluded that the robotic approach is noninferior compared to ORC and that RARC compared favorably to ORC in several perioperative parameters as well.

## Pelvic Lymphadenectomy

It has been well documented that PLND at the time of radical cystectomy provides prognostic information, but can also improve survival. Herr and colleagues reported that a minimum of nine LN should be retrieved as survival improved in LN-positive patients who had an increasing number of LN resected[22]. A thorough LN dissection can potentially increase the retrieval of positive LN that may help to guide adjuvant therapy. The robotic PLND is technically challenging, but following the anatomic boundaries as outlined above will allow for a thorough LN dissection.

Woods and colleagues retrospectively evaluated the PLND in 27 patients who underwent RARC at two institutions that submitted the LN in packets and used the bifurcation of the aorta as the proximal extent of dissection[5]. The average LN yield in these patients was 12.3 (range 7–20), but, interestingly, the average at one institution was 11.3 vs. 16.0 at the second institution, highlighting the central role the pathologist plays in identifying LN. The Roswell Park Cancer Institute reported LN yield results from their single institution that prospectively collected data on 47 consecutive patients who underwent RARC with extracorporeal urinary diversion and PLND[23]. The mean LN yield was 18 for the entire series, but when the cohort was divided into quintiles, these authors noted that as surgeon experience increased over time, so did the LN yield, from a mean of 13 in the first quintile to a mean of 23 in the last quintile.

Richards and coworkers evaluated the LN yield in the first 35 patients who underwent RARC with extracorporeal urinary diversion and PLND at their institution, and compared the results to the last 35 patients who underwent ORC[24]. Following the same template for robotic and open PLND with the bifurcation of the aorta as the proximal extent of dissection, the median LN yield was 16 (interquartile range [IQR] 11, 24) in the RARC group and 15 (IQR 11, 22) in the ORC group ($p = 0.5$).

Recently, the International Robotic Cystectomy Consortium (IRCC) was created to critically evaluate the results of RARC for clinically localized bladder cancer from 2003–2009. This database combined prospectively collected data from 15 academic and private institutions from around the globe. The retrospective review of the database included 527 patients, of which 437 (82.9%) had a lymphadenectomy performed (defined as ≥10 nodes sampled)[25]. A mean of 17.8 (range 0–68) LN were sampled; however, there did appear to be a learning curve associated with the performance of PLND at the time of RARC as high-volume surgeons (>20 cases) were almost three times more likely to perform PLND than lower-volume surgeons.

## Oncologic and Functional Outcomes

Muscle-invasive urothelial bladder cancer is an aggressive and potentially lethal disease that depends on sound oncologic surgical principles to optimize survival[26]. Early critics of RARC have been skeptical as no long-term >5-year oncologic follow-up has been reported for RARC. Dasgupta and colleagues reported short- to medium-term oncologic and functional outcomes in 20 patients who underwent RARC at their institution, with median follow-up of 23 months (range 7–44 months)[27]. They reported an overall and disease-free survival of 95 and 90%, respectively, in this highly selected cohort. In regards to functional outcomes, one of the three patients who underwent ECRONB developed a neovesicourethral stricture at 3 months that required endoscopic dilation. The authors did not include detailed data on erectile function, but noted that three of four patients who were potent preoperatively and who underwent nerve sparing were potent with phosphodiesterase-5 inhibitors postoperatively.

In the largest single-institution series to date, Pruthi et al. reported the surgical and pathologic outcomes in 100 consecutive patients who underwent RARC from 2006–2009[28]. In the entire cohort, 13% of patients were noted to have extravesical disease and 20% had LN metastasis on final pathology. With an average clinical follow-up of 21.2 months (range 5–44), the overall and disease-specific survival was 91 and 94%, respectively, mirroring the results reported by Dasgupta et al. Another study from two institutions evaluated intermediate oncologic efficacy in 59 patients who underwent RARC for bladder cancer with curative intent[29]. At a mean follow-up of 25 months (range 6–49), the overall and disease-specific survival rates at 12 months were 82%, and the overall and disease-specific survival rates at 36 months were 69 and 72%, respectively. These short- to intermediate-term results are comparable to survival rates from ORC, which is encouraging, but ultimately requires longer-term follow-up and larger numbers of patients.

## Learning Curve

Laparoscopic radical cystectomy has not achieved widespread acceptance partly due to the technical expertise required; however, RARC has been reported to have a relatively short learning curve. Investigators from The University of North Carolina at Chapel Hill were the first to critically evaluate the learning curve of RARC using EBL, operative time, pathologic outcomes, and complication rate as learning curve metrics[30]. The first 50 patients who underwent RARC at this institution were included in this analysis. After the 20$^{th}$ case, operative times and EBL seemed to reach a plateau, suggesting that a surgeon with prior experience with robotic prostatectomy can reach a level of proficiency by the 20$^{th}$ case. The LN yield, positive margin rate, and complication rate were not compromised during the learning curve phase in this cohort. This is in contrast to the RARC series reported by Guru and colleagues, which noted that as experience increased, so did the mean LN yield, from 13 to 23, from the first to fifth quintile in a cohort of 47 patients[23].

The learning curve results were recently published from the IRCC database that included 496 patients who underwent RARC by 21 surgeons at 14 institutions from 2003–2009[31]. Using predetermined cutoff points for operative time, LN yield, EBL, and margin positivity, the authors concluded that by the surgeon’s 30$^{th}$ case, he/she was able to achieve an acceptable level of proficiency with this technically challenging procedure.

## Complications

The morbidity of ORC has been well documented, with an incidence of early complications occurring within 30 days of surgery ranging between 20–57%[32,33]. The majority of ORC series that reported on complications failed to use a formal complication reporting system, which may have led to the great variability in the incidence of complications reported. Shabsigh and colleagues retrospectively reviewed the Memorial Sloan-Kettering Cancer Center radical cystectomy database and used a complication grading system to critically evaluate all complications within 90 days of surgery in 1,142 consecutive patients. They noted that 64% of patients experienced a complication with a 30-day mortality rate of 1.5%[34]. One of the hopes for RARC would be to decrease this remarkably high complication rate.

Kauffman et al. specifically looked at the complication rate of RARC in a single-surgeon consecutive series of 79 patients using two standardized reporting methods (Memorial Sloan-Kettering Cancer Center and Modified Clavien)[35]. In this series, 49% of patients had a complication at 90 days, with 16% being "high grade". Of note, independent risk factors for high-grade complications on multivariate analysis included age ≥65, operative blood loss ≥500 mL, and intraoperative intravenous fluids of >5000 mL. This same group later went on to compare the complication rate from RARC (n = 83) to ORC (n = 104) in 187 consecutive patients[36]. Complications were compiled prospectively using the Modified Clavien system, and recorded at 30 and 90 days. The results showed a nonstatistically significant trend to more overall complications at 90 days in the ORC group compared to the RARCgroup (62 vs. 48%, $p = 0.07$); however, there were significantly more major complications in the ORC group (31 vs. 17%, $p = 0.03$) at 90 days. Furthermore, on logistic regression analysis, RARC was an independent predictor for fewer complications. Selection bias, team approach, and surgeons having prior experience with open or laparoscopic radical cystectomy before embarking on RARC could have influenced the trend towards fewer overall complications in the RARC group. These results are encouraging and may help to increase the adoption of RARC in high-volume centers.

## FUTURE DIRECTIONS

While RARC is still in its infancy, with promising early and intermediate perioperative, functional, and oncologic outcomes, the future of RARC as a treatment modality for bladder cancer depends on the results from long-term oncologic follow-up; for instance, the IRCC recently reported the incidence of positive surgical margins from their database and noted a 39% positive margin rate in patients with pT4 disease[37]. In comparison, the positive margin rate for pT4 disease was 24% in a multicenter study involving >4,400 patients who underwent ORC for bladder cancer[38]. The increased incidence of positive margins from RARC for extravesical disease will require further study with larger numbers of patients and longer-term follow-up for disease-specific survival and overall survival. Furthermore, there is a paucity of data in the literature evaluating long-term complications as a notable proportion may occur at >30 days postcystectomy[35]. Additionally, the literature is lacking in regards to functional outcomes and quality of life (QoL) outcome measures, although one study reported prompt return of QoL to baseline levels at 6 months post-RARC utilizing the Functional Assessment of Cancer Therapy – Bladder questionnaire (FACT-BL)[39].

Also at this point in time, total intracorporeal urinary diversion is evolving, but a majority of centers are focusing on extracorporeal urinary diversion utilizing the incision used for retrieval of the specimen. The authors have experience of both techniques and do not perceive any outstanding advantages of total intracorporeal urinary diversion over extracorporeal urinary diversion. Total intracorporeal urinary diversion would certainly be advantageous in a female patient where the specimen can be retrieved transvaginally. At this point in time, prior marking of the ileocecal junction and ileal conduit, and transferrance of the left ureter to the right, all leads to a smaller incision and faster extracorporeal urinary diversion, therefore reducing operative time and the impact of Trendelenburg position and pneumoperitoneum, especially in the elderly population. Furthermore, intracorporeal urinary diversion is technically demanding and can only be performed by select experienced surgeons at select high-volume centers in a select subset of patients.

With health care reform on the horizon, the financial cost of RARC will also require critical evaluation as a single-institution study noted that RARC was associated with an increased cost of $1,650 compared to ORC in the perioperative setting[40].

## CONCLUSIONS

RARC is technically feasible and has been shown to have acceptable short- to intermediate-term oncologic outcomes. This technique is gaining popularity due to the acceptable learning curve for surgeons adept at open or laparoscopic radical prostatectomy, and who have experience with robotic radical prostatectomy. Bladder cancer is a potentially lethal malignancy afflicting elderly patients with multiple medical comorbidities, therefore necessitating long-term follow-up of oncologic and functional outcomes, which should be forthcoming in the years to come.
